# Cystoid Macular Edema in Bietti's Crystalline Retinopathy

**DOI:** 10.1155/2014/964892

**Published:** 2014-05-11

**Authors:** Ali Osman Saatci, Hasan Can Doruk, Aylin Yaman

**Affiliations:** Department of Ophthalmology, Dokuz Eylul University, Mithatpasa Caddesi, 35300 Izmir, Turkey

## Abstract

A 27-year-old man with progressive bilateral visual decline was diagnosed to have Bietti's crystalline dystrophy (BCD). Fluorescein angiography revealed bilateral petaloid type late hyperfluorescence implicating concurrent cystoid macular edema (CME). Optical coherence tomography exhibited cystoid foveal lacunas OU. During the follow-up of six years, intraretinal crystals reduced in amount but CME persisted angiographically and tomographically. CME is among the rare macular features of BCD including subfoveal sensorial detachment, subretinal neovascular membrane, and macular hole.

## 1. Introduction

In 1937, Bietti [1] described three cases characterized by glistening, yellow-white intraretinal crystals in the posterior pole, atrophy of the retinal pigment epithelium, choroidal sclerosis, crystals in the superficial, paralimbal cornea, and onset in the third decade of life.

Bietti's crystalline dystrophy (BCD) is likely related to aberrant oxidation of cellular lipid metabolism [2] which is caused by mutations of the CYP4V2 gene, a member of the cytochrome P450 genes [3], and the dystrophy can be familial [4, 5]. Hallmark of the disease is the presence of intraretinal crystals mostly located paracentrally and the crystals particularly lie at the transition zone between relatively normal and atrophic retinal pigment epithelium [6–9]. These crystals progressively disappear within time and are replaced by areas of chorioretinal atrophy. The disease has not been observed in childhood and affected patients experience visual acuity deterioration, night blindness, and paracentral scotoma between the second and fourth decades of life. Visual decrease may even lead to legal blindness by the fifth and sixth decades of life. Lai et al. [10] evaluated the genotype-phenotype analysis of BCD in a group of 18 Chinese patients in 13 families and showed that BCD patients with homozygous IVS6-8del17 bp/insGC or compound heterozygous IVS6-8del17 bp/insGC and IVS8-2A_G mutations appeared to have a more severe disease phenotype based on electrophysiological testing. On the other hand, Rossi et al. [11] described the clinical and genetic features of 15 Italian patients with BCD and demonstrated seven new mutations illustrating the large range of genotypic and phenotypic variability highlighting the lack of a clear genotype-phenotype correlation.

Macular complications are rarely encountered in patients with BCD. We hereby report a case with BCD and bilateral persistent cystoid macular edema with a follow-up of six years.

## 2. Report of a Case 

**Figure 1:**
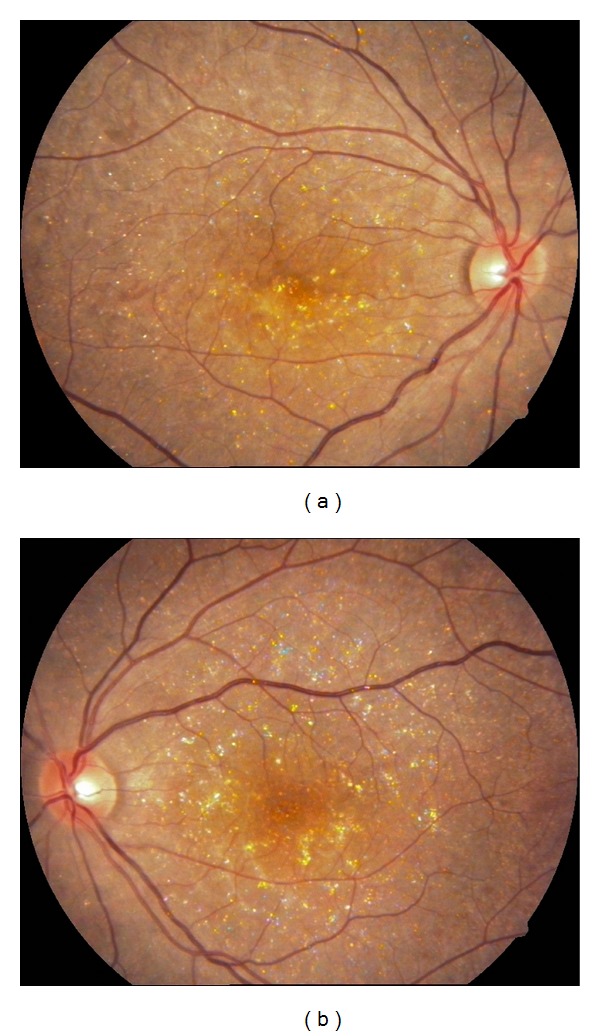
Color fundus pictures depicting the intraretinal crystals. (a) Right eye and (b) left eye.

**Figure 2:**
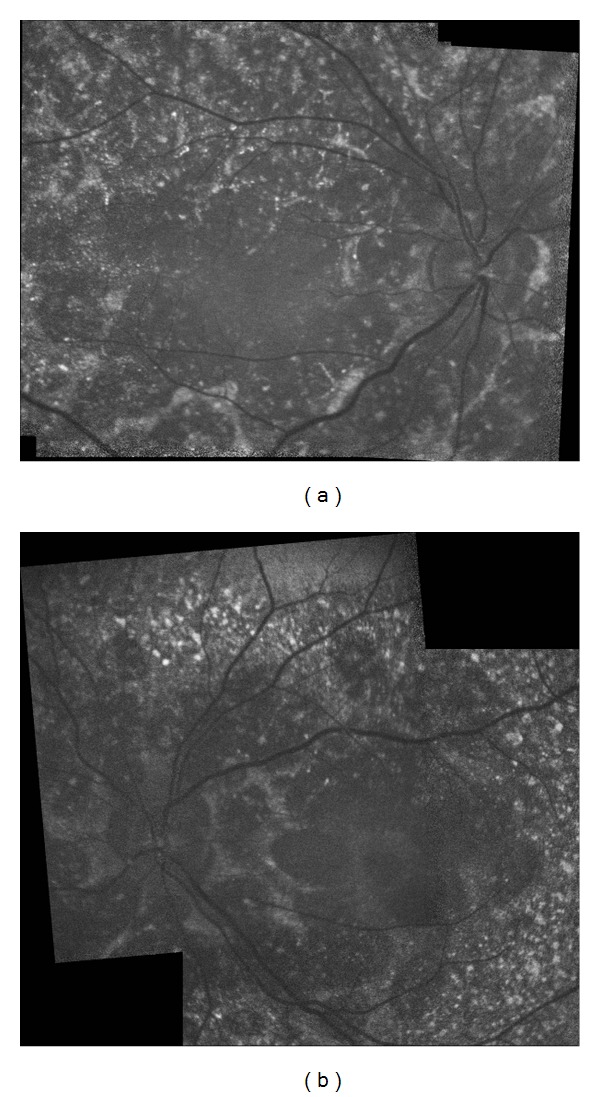
Composite fundus autofluorescent pictures demonstrating the central hypoautofluorescent areas and dot-like hyperautofluorescent spots surrounding the macula. (a) Right eye and (b) left eye.

**Figure 3:**
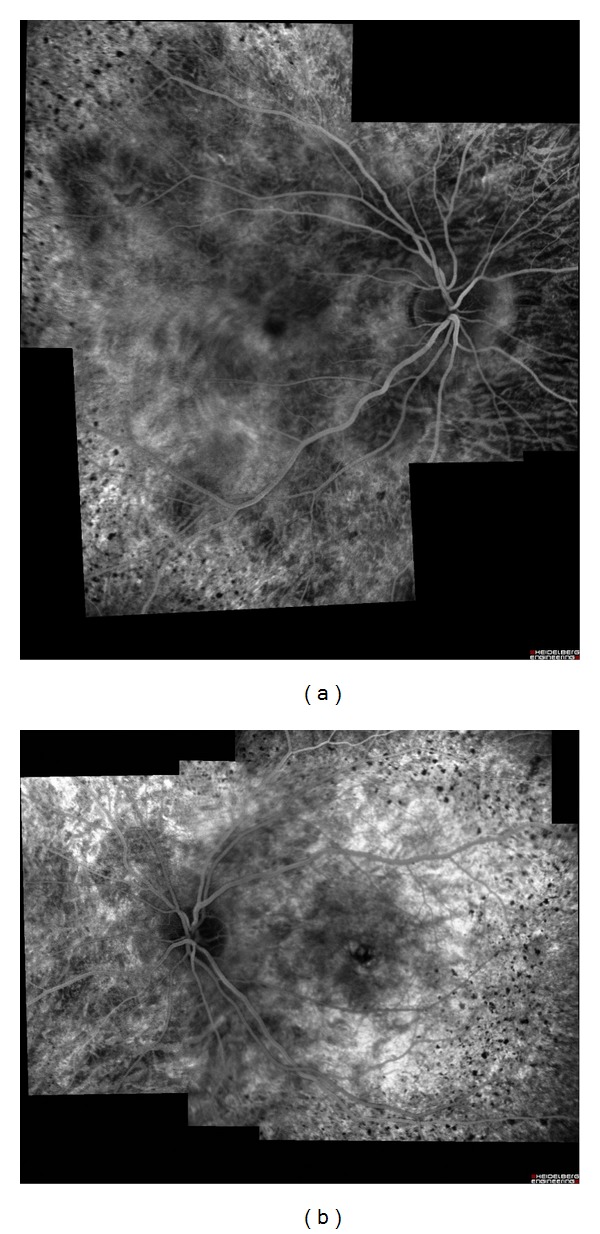
Fluorescein angiogram, composite picture, late venous phase showing cystoid macular edema, blocking effect of intraretinal crystals, and slight chorioretinal atrophy. (a) Right eye (b) and left eye.

**Figure 4:**
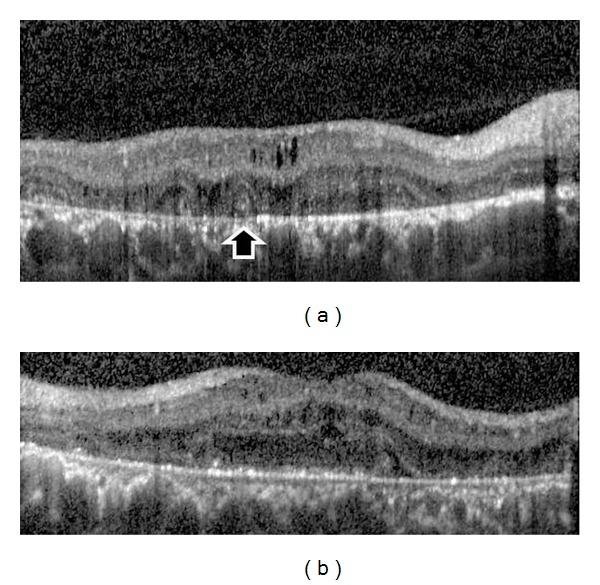
(a) SD-OCT of the right eye depicting the intraretinal cysts, outer segment tubulation (black arrow), and vitreomacular adhesion. (b) SD-OCT of the left eye demonstrating the intraretinal cysts.

A 27-year-old otherwise healthy man was examined by us for bilateral progressive visual loss of long duration. There was no family history of consanguinity or similarly affected family members. On examination, best-corrected visual acuity was 20/40 OD and 20/50 OS. Examination of the anterior segment was normal with no concurrent corneal crystals. Intraocular pressures were 16 mm Hg OU. Fundus examination showed refractile intraretinal crystals in the posterior pole and the midperiphery associated with slight RPE atrophy (Figures 1(a) and 1(b)). Fundus autofluorescence imaging exhibited areas of low autofluorescent signal corresponding to areas of retinal atrophy at the posterior pole with multiple dotty hyperautofluorescence around the macula OU (Figures 2(a) and 2(b)). Fluorescein angiography exhibited petaloid type late foveal hyperfluorescence implicating cystoid macular edema OU (Figures 3(a) and 3(b)). SD-OCT exhibited cystoid changes bilaterally (Figures 4(a) and 4(b)).

**Figure 5:**
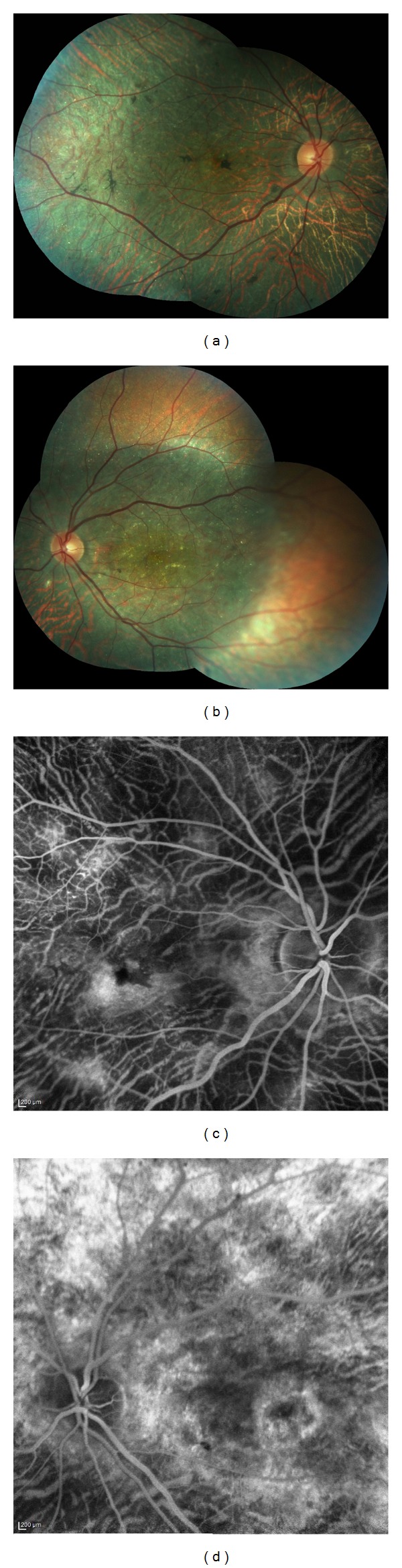
Color fundus pictures showing reduced numbers of intraretinal crystals and increased chorioretinal atrophy with some retinal pigment epithelial hyperplasia six years after the initial presentation. (a) Right eye and (b) left eye. Angiographic appearance of persistent cystoid macular edema. (c) Right eye and (d) left eye.

**Figure 6:**
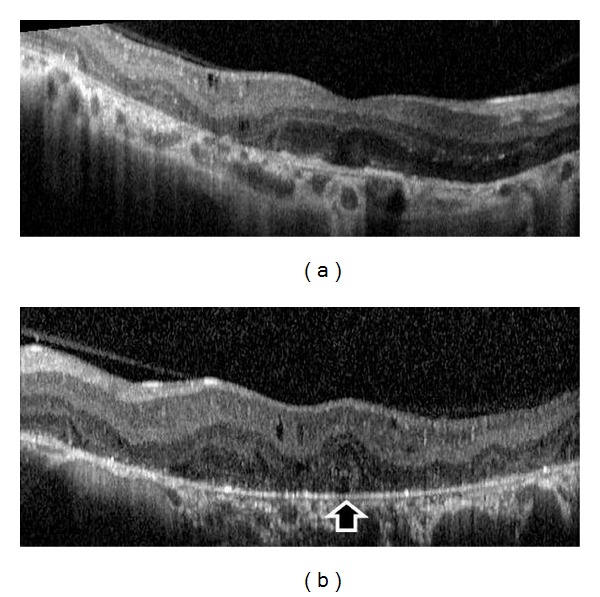
Optic coherence tomography. (a) Right eye, cystoid changes, and concurrent vitreomacular adhesion. (b) Left eye, cystoid changes, outer retinal tubulation (black arrow), and vitreomacular adhesion.

The diagnosis was BCD with bilateral cystoid macular edema. No treatment was given. The patient was reexamined six years later. This time, visual acuities were 20/80 OD and 20/100 OS. Ophthalmologic findings were more pronounced when compared to initial ophthalmological examination with marked chorioretinal atrophy, reduced numbers of crystals, and the occurrence of patchy retinal pigment epithelial hyperplasia. (Figures 5(a), 5(b), 5(c), and 5(d)) CME was still persisting OU tomographically (Figures 6(a) and 6(b)).

## 3. Discussion

Cone and rod photoreceptors are equally affected in many cases with BCD and therefore central vision drops even at early stages of the disease in some cases [2]. In addition to evolving disease process already compromising the macular function several rare macular changes may further decrease the visual acuity. Subfoveal neurosensory detachment [12], subretinal neovascular membrane [13–15], and macular hole formation [16, 17] are among the previously noted macular changes in eyes with BCD.

Cystoid macular edema (CME) represents a known complication in patients with some hereditary retinal degenerations such as retinitis pigmentosa, X-linked retinoschisis, enhanced cone syndrome, choroideremia, and gyrate atrophy and is characterized by a localised expansion of the macular intracellular and/or extracellular space [18, 19]. Various pathogenetic mechanisms have been offered to explain the cystic macular lesions in retinal dystrophies including blood-retinal barrier impairment and tangential vitreous traction [19]. Spalton et al. [20] suggested that macular edema might be due to an inflammatory response seen in many types of tapetoretinal degenerations against actively degenerating photoreceptors and retinal pigment epithelium. Heckenlively et al. [21] speculated that retinitis pigmentosa-related macular edema might have an underlying autoimmune process. Oral or topical carbonic anhydrase inhibitors, intravitreal corticosteroids (triamcinolone acetonide or dexamethasone implant), or anti-VEGF drugs were administered with some success in cases with macular edema related to retinitis pigmentosa [19, 22]. To our best knowledge, CME was reported only in two cases with BCD. Parravano et al. [23] reported a 39-year-old man with BCD and concurrent CME. Despite having a good visual acuity optical coherence tomography demonstrated the presence of bilateral cystoid edema. However, no angiographic findings were present in the paper. Broadhead and Chang [24] very recently described a 32-year-old male of Maltese heritage with BCD and CME. After initiation of oral acetazolamide treatment (500 mg/day) visual acuity and macular anatomy improved bilaterally within a month. In our case, there was a petaloid type bilateral macular leakage suggesting the CME. Edema almost stayed the same during a follow-up of six years OU. Petaloid type of fluorescein leakage might imply that edema has a vascular component. Notingly, vitreomacular adhesion was also present which might also contribute to CME formation in our case. No treatment was given for the macular edema in the present case.

In light of our observation, we believe that CME should be looked for in eyes with BCD besides the other rarely reported macular changes. However, therapy of CME in BCD is uncertain.

## References

[1] Bietti GB (1937). Uber familiares vorkommen von “Retinitis punctata albescens” (verbunden mit “Dystrophia marginalis cristallinea corneae” ), Glitzern des Glasköpers und anderen degenerativen Augenveranderungen. *Klinische Monatsblatter fur Augenheilkunde's*.

[2] Furusato E, Cameron JD, Chan CC (2010). Evolution of cellular inclusions in Bietti's Crystalline Dystrophy. *Journal of Ophthalmology and Eye Diseases*.

[3] Li A, Jiao X, Munier FL (2004). Bietti crystalline corneoretinal dystrophy is caused by mutations in the novel gene CYP4V2. *American Journal of Human Genetics*.

[4] Chan W-M, Pang C-P, Leung ATS, Fan DSP, Cheng ACK, Lam DSC (2000). Bietti crystalline retinopathy affecting all 3 male siblings in a family. *Archives of Ophthalmology*.

[5] Saatci AO, Yaman A, Öner FH, Ergin MH, Çingil G (2002). Indocyanine green angiography in Bietti’s crystalline retinopathy. *Canadian Journal of Ophthalmology*.

[6] Bernauer W, Daicker B (1992). Bietti’s corneal-retinal dystrophy. A 16-year progression. *Retina*.

[7] Kaiser-Kupfer MI, Chan C-C, Markello TC (1994). Clinical biochemical and pathologic correlations in Bietti’s crystalline dystrophy. *American Journal of Ophthalmology*.

[8] Oner FH, Saatci AO, Ergin M, Cingil G (2002). Bietti'nin Kristalin Retinopatisi. *Türk Oftalmoloji Gazetesi*.

[9] Mansour AM, Uwaydat SH, Chan C-C (2007). Long-term follow-up in Bietti crystalline dystrophy. *European Journal of Ophthalmology*.

[10] Lai TYY, Tsz KN, Tam POS (2007). Genotype-phenotype analysis of Bietti’s crystalline dystrophy in patients with CYP4V2 mutations. *Investigative Ophthalmology and Visual Science*.

[11] Rossi S, Testa F, Li A (2013). Clinical and genetic features in Italian Bietti crystalline dystrophy patients. *British Journal of Ophthalmology*.

[12] Padhi TR, Kesarwani S, Jalali S (2011). Bietti crystalline retinal dystrophy with subfoveal neurosensory detachment and congenital tortuosity of retinal vessels: case report. *Documenta Ophthalmologica*.

[13] Le Tien V, Atmani K, Querques G, Massamba N, Souied EH (2010). Ranibizumab for subfoveal choroidal neovascularization in Bietti crystalline retinopathy. *Eye*.

[14] Gupta B, Parvizi S, Mohamed MD (2011). Bietti crystalline dystrophy and choroidal neovascularisation. *International Ophthalmology*.

[15] Nachiappan K, Krishnan T, Madhavan J (2012). Ranibizumab for choroidal neovascular membrane in a rare case of Bietti’s crystalline dystrophy: a case report. *Indian Journal of Ophthalmology*.

[16] Bagolini B, Ioli-Spada G (1968). Bietti’s tapetoretinal degeneration with marginal corneal dystrophy. *American Journal of Ophthalmology*.

[17] Saatci AO, Yaman A, Berk AT, Söylev MF (1997). Macular hole formation in Bietti’s crystalline retinopathy: a case report. *Ophthalmic Genetics*.

[18] Sahel J, Bonnel S, Mrejen S, Paques M (2010). Retinitis pigmentosa and other dystrophies. *Developments in Ophthalmology*.

[19] Salvatore S, Fishman GA, Genead MA (2013). Treatment of cystic macular lesions in hereditary retinal dystrophies. *Survey of Ophthalmology*.

[20] Spalton DJ, Bird AC, Cleary PE (1978). Retinitis pigmentosa and retinal oedema. *British Journal of Ophthalmology*.

[21] Heckenlively JR, Jordan BL, Aptsiauri N (1999). Association of antiretinal antibodies and cystoid macular edema in patients with retinitis pigmentosa. *American Journal of Ophthalmology*.

[22] Saatci AO, Selver OB, Seymenoglu G, Yaman A (2013). Bilateral intravitreal dexamethasone implant for retinitis pigmentosa-related macular edema. *Case Reports in Ophthalmology*.

[23] Parravano M, Sciamanna M, Giorno P, Boninfante A, Varano M (2012). Bietti crystalline dystrophy: a morpho-functional evaluation. *Documenta Ophthalmologica*.

[24] Broadhead GK, Chang AA (2014). Acetazolamide for cystoid macular oedema in Bietti Crystalline Retinal dystrophy. *Korean Journal of Ophthalmology*.

